# Taft’s Pluggers

**Published:** 1858-04

**Authors:** Jno. T. Toland

**Affiliations:** 38 West Fourth Street, Cincinnati, O.


### DR. TAFT'S PLUGGERS FOR CRYSTAL GOLD AND ADHESIVE FOIL.

For a description of Dr. Taft's mode of filling, we refer to the March number of the "Dental Register," which we presume every one subscribes for—if they do not, they should do so at once,—as the best Dental Journal in existence!

Dr. Taft's set of pluggers for crystal gold and annealed gold foil, consists of 16 instruments, all the *points* with a less variety of sizes, may be embraced in 1 dozen, or by multiplying the *sizes*, which would be advisable, may with propriety be increased to 2 dozen, prices same as Blakesley's patterns. We have been making only one style, viz: ¼ in. octagon steel, with alternate sides, file cut, and polished, price five dollars per dozen; but in these as with any other patterns, any style will be furnished, as the handle and style regulates the price.

Instruments with proper shapes and finely and deeply serrated points, are of the first importance; *on them* depends the *perfection* of the work.

Our prices for these instruments are as follows:

| | | | |
|---|---|---|---|
| ¼ | In. Octagon steel plain, .................. | 2,50 | per dozen. |
| ¼ | " " " with knobs, ....... | 3,50 | " |
| ¼ | " " file cut " " ........ | 4,50 | " |
| ¼ | " " alternate sides polished,.... | 5,00 | " |
| ½ | " ebony albata ferrules,.......... | 6,50 | " |
| ½ | " " silver " ............ | 8,50 | " |
| ½ | " ivory albata " ............ | 9,00 | " |
| ½ | " " silver " ............ | 11,00 | " |
| ⅝ | " ebony albata " ............ | 8,00 | " |
| ⅝ | " " silver " ............ | 11,00 | " |
| ⅝ | " ivory albata, " ............ | 10,50 | " |
| ⅝ | " " silver " ............ | 13,50 | " |

And other sizes in proportion. We are prepared and will be pleased to supply the profession and dealers with these *valuable* instruments.

JNO. T. TOLAND,
38 West Fourth Street, Cincinnati, O.

